# Exploration of the Antibacterial and Anti-Inflammatory Activity of a Novel Antimicrobial Peptide Brevinin-1BW

**DOI:** 10.3390/molecules29071534

**Published:** 2024-03-29

**Authors:** Zhizhi Chen, Lei Wang, Dongxia He, Qi Liu, Qinqin Han, Jinyang Zhang, A-Mei Zhang, Yuzhu Song

**Affiliations:** 1 Research Center of Molecular Medicine of Yunnan Province, Faculty of Life Science and Technology, Kunming University of Science and Technology, Kunming 650504, China; chenzhizhi@stu.kust.edu.cn (Z.C.); 20222118052@stu.kust.edu.cn (L.W.); hedongxia@stu.kust.edu.cn (D.H.); liuqi7@stu.kust.edu.cn (Q.L.); qqhan10@kust.edu.cn (Q.H.); jyzhang@kust.edu.cn (J.Z.); zam1980@kust.edu.cn (A.-M.Z.); 2 School of Medicine, Kunming University of Science and Technology, Kunming 650504, China

**Keywords:** antimicrobial peptides, Brevinin-1, LPS, anti-inflammation

## Abstract

Antibiotic resistance has emerged as a grave threat to global public health, leading to an increasing number of treatment failures. Antimicrobial peptides (AMPs) are widely regarded as potential substitutes for traditional antibiotics since they are less likely to induce resistance when used. A novel AMP named Brevinin-1BW (FLPLLAGLAASFLPTIFCKISRKC) was obtained by the Research Center of Molecular Medicine of Yunnan Province from the skin of the *Pelophylax nigromaculatus*. Brevinia-1BW had effective inhibitory effects on Gram-positive bacteria, with a minimum inhibitory concentration (MIC) of 3.125 μg/mL against *Enterococcus faecalis* (ATCC 29212) and 6.25 μg/mL against both *Staphylococcus aureus* (ATCC 25923) and multidrug-resistant *Staphylococcus aureus* (ATCC 29213) but had weaker inhibitory effects on Gram-negative bacteria, with a MIC of ≥100 μg/mL. Studies using scanning electron microscopy (SEM) and flow cytometry have revealed that it exerts its antibacterial activity by disrupting bacterial membranes. Additionally, it possesses strong biofilm inhibitory and eradication activities as well as significant lipopolysaccharide (LPS)-binding activity. Furthermore, Brevinin-1BW has shown a significant anti-inflammatory effect in LPS-treated RAW264.7 cells. In conclusion, Brevinin-1BW is anticipated to be a promising clinical agent with potent anti-Gram-positive bacterial and anti-inflammatory properties.

## 1. Introduction

Numerous prevalent diseases, such as gastrointestinal infections, urinary tract infections, otitis media, osteomyelitis, and septic arthritis, are brought on by pathogenic bacteria infections [1,2,3,4,5,6,7,8]. *E. coli*, *P. aeruginosa*, and *S. aureus* are some examples of common pathogenic bacteria; antibiotics are one of the main drugs for treating bacterial infections. Antibiotics exhibit considerable effectiveness in terms of a spectrum of activity and bactericidal effects, swiftly eliminating infections and improving patient recovery rates. However, the overuse and misuse of antibiotics have led to the problem of antibiotic resistance [9,10,11,12,13], rendering some antibiotics gradually ineffective, posing a serious challenge to global health. Hence, there is an urgent need to develop new antimicrobial agents. Antimicrobial peptides (AMPs) are natural immune defense molecules with various unique biological characteristics, including broad-spectrum antimicrobial activity, bactericidal effects, and activity against drug-resistant bacteria [14,15,16,17]. AMPs represent a potential effective alternative [18,19,20]. AMPs have multiple mechanisms of action against bacteria, achieving antimicrobial effects by disrupting bacterial cell membranes [21,22,23], interfering with metabolic pathways, and regulating immune responses [24,25,26,27]; therefore, they are less likely to result in the evolution of drug-resistant strains. Additionally, antimicrobial peptides cause minimal damage to host cells and enhance host immune function, aiding in disease recovery and maintaining physiological functions [28,29]. Furthermore, antimicrobial peptides have potential effects such as anti-inflammatory properties [30,31], anticancer properties [32], and insulin-release promotion [33,34,35], indicating their potential as antibiotic alternatives. However, compared to antibiotics, the pharmacological properties of antimicrobial peptides are still under investigation, and their clinical applications are in the early stages, necessitating further research and development for widespread use. In summary, antibiotics have extensive applications and experience in treating bacterial infections, but the issue of resistance is increasingly prominent. Antimicrobial peptides have the potential to be a new class of drugs to overcome resistance, but their clinical application requires further research and development. In the future, combining the advantages of both may provide more choices and solutions for treating infectious diseases.

Belonging to the Brevinin superfamily, Brevinin-1 peptide is the most prevalent AMP derived from amphibians [36]. The Brevinin-1 family of AMPs is a class of small molecule peptides, typically containing 24 amino acids, that primarily exert bactericidal effects by disrupting bacterial cell membranes [37,38]. AMPs of the Brevinin-1 family can traverse membrane surfaces and exhibit significant hydrophilicity and hydrophobicity. Brevinin-1 peptides have been found to exist in an amphiphilic-helical structure in a hydrophobic membrane-simulated environment; these can bind to and alter the cell membrane to disrupt the phospholipid bilayer and cause bacterial death as well as modulate ions and interact with the hydrogen bonds that are each present in the cell membrane [38,39].

Based on the above background, a novel peptide, named Brevinin-1BW, was investigated for its activity and showed great potential against Gram-positive bacteria. The secondary structure, physicochemical properties, and three-dimensional structure of Brevinin-1BW were predicted with online analysis tools, and the peptide was successfully synthesized via solid phase synthesis. Further experiments were performed to evaluate the antimicrobial and hemolytic activities of Brevinin-1BW as well as its inhibition against Gram-positive bacteria. In particular, this study revealed the potent antibacterial mechanism of Brevinin-1BW by highly penetrating the bacterial cell membrane using a cell membrane permeability assay and time-killing curve assay. In addition, the cytotoxicity of Brevinin-1BW was extensively evaluated, including studies on a variety of cancer cells and non-cancer cells, to ensure its safety for potential clinical application. The research of Brevinin-1BW in the antibacterial field not only focuses on the evaluation of antibacterial activity but also explores its biofilm inhibition ability and lipopolysaccharide (LPS)-binding efficiency. These properties make Brevinin-1BW show great potential in inhibiting biofilm-associated infections and inflammation caused by Gram-positive bacteria. Overall, Brevinin-1BW, as an AMP with potent antimicrobial and anti-inflammatory properties, provides a valuable basis for the development of new therapeutic strategies, contributing a new option in the battle against Gram-positive bacteria.

## 2. Results

### 2.1. Identification and Synthesis of Brevinin-1BW

**Figure 1:**
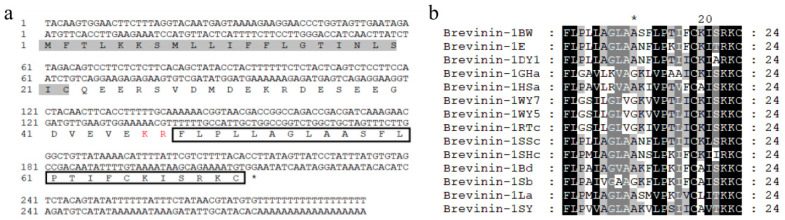
Sequence characterization of Brevinin-1BW. (**a**) cDNA and predicted amino acid sequence of Brevinin-1BW. The signal peptide is marked with a gray background, the KR protease cleavage site is marked in red font, the mature peptide sequence is boxed, and the stop codon is indicated by an asterisk (*). (**b**) Amino acid sequence comparison of mature short peptide Brevinin-1BW with different Brevinin-1 family peptides; highly conserved amino acid residues are indicated by the black background.

Previously, the full-length cDNA encoding Brevinin-1BW was successfully cloned from a cDNA library derived from skin secretions of *Pelophyllax nigromaculatus* by Research Center of Molecular Medicine of Yunnan Province. The nucleotide sequence and the post-translational open reading frame amino acid sequence show that the predicted AMP precursor consists of 72 amino acid residues, containing a 22-residue signal peptide, a 23-residue acidic structural domain, a classical protease cleavage KR site, and a 24-residue mature peptide segment (Figure 1a). The NCBI Basic Local Alignment Search Tool (BLAST) analysis showed that the predicted protein precursor sequences share similarity with the Brevinin-1 family peptides; they have highly similar conserved structural amino acids, and like other members of the Brevinin-1 family, the C-terminus of the peptide contains a “Rana box” structure, which consists of a disulfide bond between two Cys residues at the C-terminus (Figure 1b). Therefore, we determined that this new peptide belongs to the Brevinin-1 family and named it Brevinin-1BW accordingly.

### 2.2. Structural Analysis of Brevinin-1BW

**Figure 2:**
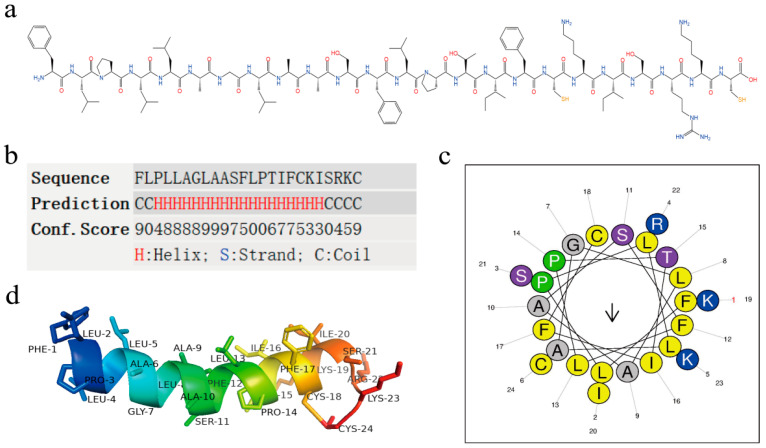
Structural parameters and structure prediction of Brevinin-1BW. (**a**) Chemical structure of Brevinin-1BW; (**b**) The α-helix structure in the secondary structure of Brevinin-1BW; (**c**) Helical wheel projection of Brevinin-1BW with arrows indicating hydrophobic surface. Numbers represent the sequence and position of amino acids; (**d**) 3D structure projection of Brevinin-1BW, the model was generated by AlphaFold2 server, and the visualization of the structure was performed using PyMOL2.5 (Schrödinger Inc., San Diego, CA, USA). Numbers represent the sequence and position of amino acids.

We predicted and analyzed the chemical structure of Brevinin-1BW (Figure 2a). Its mature peptide contains 24 amino acids, including three essential amino acids (Arg + Lys) and no acidic amino acids (Asp + Glu), with a theoretical molecular weight of 2610.26 Da and an isoelectric point of 9.5. To further investigate the secondary structure of Brevinin-1BW and its structural parameters, the online tools Heliquest and I-TASSER (Figure 2b,c) were used in the analysis to predict its secondary structure. The results showed that the AMP contains a large proportion of α-helical structures with hydrophobic surfaces consisting of I, A, L, I, L, A, C, F, and A (Table 1). The three-dimensional model of Brevinin-1BW was predicted by the Alphafold2 server (Figure 2d), which corresponded to the secondary structure prediction results, and the AMP tended to form a highly α-helical structure.

### 2.3. Antibacterial Activity Analysis

The MIC of Brevinin-1BW against 12 strains was determined by the two-fold dilution method, and the results are shown in Table 2. Brevinin-1BW exhibited significant inhibitory activity against Gram-positive bacteria, where its MIC against *E. faecalis* (*ATCC* 29212) was only 3.125 μg/mL. This was followed by the complete inhibition of *S. saprophyticus* (*ATCC* BAA750), *S. aureus* (MDR *ATCC* 29213), and *S. aureus* (*ATCC* 25923) at 6.25 μg/mL. We also selected two Gram-positive clinical strains, against which Brevinin-1BW showed good antibacterial activity, with a MIC of 12.5 μg/mL. However, Brevinin-1BW did not show obvious antibacterial activity against Gram-negative bacteria. Surprisingly, Brevinin-1BW at 50 μg/mL inhibited *C. koseri* (CI 1611SED223). Brevinin-1BW also showed excellent inhibitory activity against multidrug-resistant *S. aureus*. The MBC of Brevinin-1BW against the tested Gram-positive bacteria mainly ranged between 6.25–50 μg/mL. At 6.25 μg/mL, Brevinin-BW reduced the viability of *S. aureus*, *E. faecalis*, and *S. saprophyticus* (*ATCC* BAA750) by 99.9%. Interestingly, the MIC of Brevinin-1BW against *S. aureus* was the same as the MBC, which suggests that the mechanism by which Brevinin-1BW inhibits *S. aureus* involves its direct killing. Unfortunately, the MBC values of Brevinin-1BW against Gram-negative bacteria were all above 100 μg/mL.

### 2.4. Time-Killing Assay

**Figure 3:**
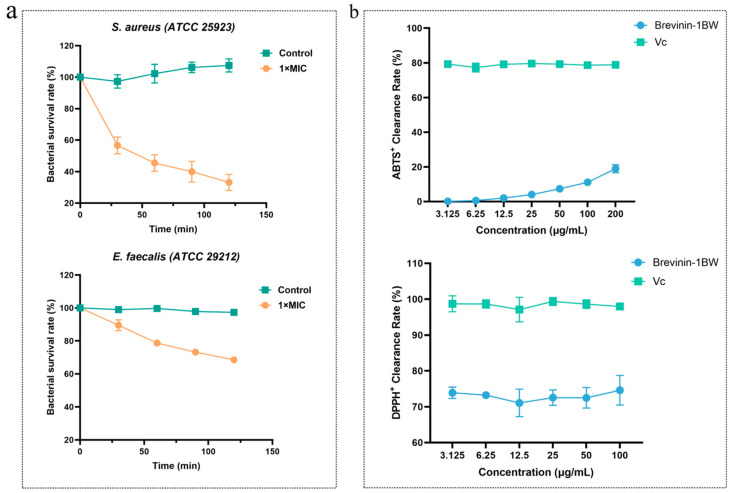
Time-killing curve and antioxidant activity of Brevinin-1BW. (**a**) The time-killing curves of Brevinin-1BW against *S. aureus* and *E. faecalis* at 1 × MIC; (**b**) Clearance of ABTS^+^ and DPPH^+^ by Brevinin-1BW at different concentrations. Error bars indicate three replicate experiments.

To evaluate the efficiency of the antibacterial function of Brevinin-1BW, the bactericidal rate of Brevinin-1BW against the strains was assessed with the time-killing curve. Both *S. aureus* and *E. faecalis* were selected as indicator strains according to the MIC of the Brevinin-1BW AMP; the results are shown in Figure 3a. The bactericidal efficiency of Brevinin-1BW was smooth, killing 67% of *S. aureus*. Approximately 32% of *E. faecalis* were killed after 120 min, and this effect was prolonged such that the growth of colonies did not resume over time. No significant change in bacterial growth was observed in the blank control group.

### 2.5. Inhibition and Elimination of Biofilms by Brevinin-1BW

**Figure 4:**
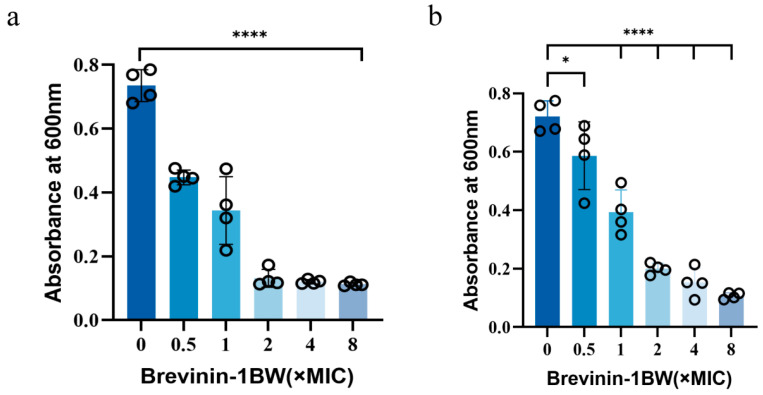
Effect of Brevinin-1BW on *S. aureus* biofilms. (**a**) Biofilm inhibitory activity of Brevinin-1BW. (**b**) Biofilm scavenging activity of Brevinin-1BW. Data represent mean SD values of three independent experiments performed in quadruplicate. The circle represents each experimental data. **** (*p* < 0.0001), * (*p* < 0.005).

Crystal violet staining and a quantitative analysis were used to investigate the inhibitory effect of Brevinin-1BW on biofilm formation and clearance of mature biofilms in *S. aureus*. When the peptide concentration was increased from 0.5 × MIC to 2 × MIC, the inhibition of biofilm formation in *S. aureus* increased from 35.76% to 83.67% (Figure 4a). At the same time, the clearance rate of the mature biofilm using Brevinin-1BW increased from 50.02% to 60.99% (Figure 4b).

### 2.6. Effect of Temperature, Serum, and pH on Brevinin-1BW

The stability of Brevinin-1BW was evaluated under different physical and chemical conditions, and the results are presented in Table 3. The MIC of Brevinin-1BW did not change significantly at the temperature range of −20 °C to 100 °C. At the same time, its antibacterial activity was still stable after incubation with a different pH. However, when Brevinin-1BW was incubated with 10% serum, its MIC became twice as high.

### 2.7. Cytotoxicity and Hemolytic Activity of Brevinin-1BW

We examined the cytotoxicity of Brevinin-1BW on HaCaT, HUVEC, HFF, RAW264.7, and 293T cells, which are slightly less toxic to normal cells than cancer cells, as shown in Table 4. In order to explore the anticancer effect of Brevinin-1BW, a CCK8 assay was used to evaluate the anticancer activity of Brevinin-1BW in six cancer cell lines A375, A549, Hela, HePG2, U-2OS, and HCT116. Overall, Brevinin-1BW showed some inhibitory effects on the proliferation of these cancer cells.

**Figure 5:**
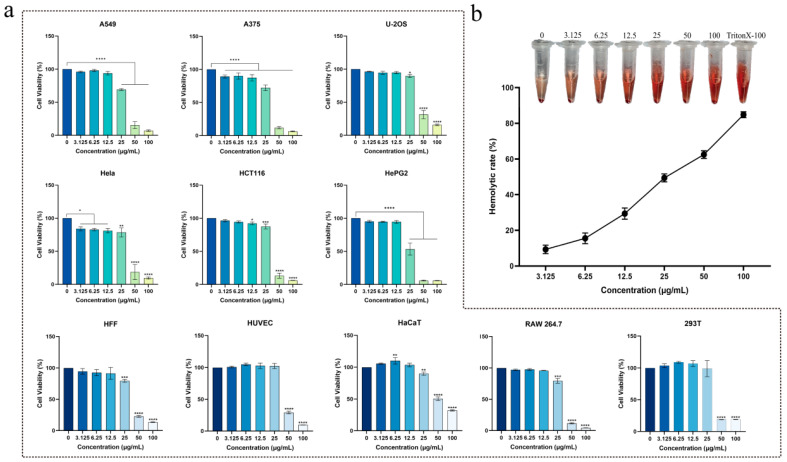
Cytotoxic and hemolytic activity of Brevinin-1BW. (**a**) Effect of Brevinin-1BW on the viability of six cancer cells and five non-cancer cells. (**b**) Hemolytic activity of different concentrations of Brevinin-1BW. **** (*p* < 0.0001), *** (*p* < 0.001), ** (*p* < 0.001), * (*p* < 0.005).

We evaluated the hemolytic activity of Brevinin-1BW using sheep erythrocytes, and like other Brevinin-1 family peptides, Brevinin-1BW exhibited robust hemolytic activity. As shown in Figure 5b, its hemolytic activity was not evident at 3.125 μg/mL, though significant hemolysis was observed at concentrations greater than 12.5 μg/mL, with an HC_50_ (50% hemolytic concentration) value of 35.8 μg/mL for Brevinin-1BW.

### 2.8. Antioxidant Activity Analysis

The antioxidant activity of Brevinin-1BW was assessed by measuring its ABTS and DPPH radical scavenging ability. The result is shown in the Figure 3b. As expected, Brevinin-1BW did not possess good antioxidant activity, and surprisingly, Brevinin-1BW achieved more than 70% scavenging of DPPH radicals at 3.125 μg/mL without concentration dependence. However, regarding ABTS radical scavenging, Brevinin-1BW performed poorly, with less than 20% scavenging at 200 μg/mL. From the experimental results, the scavenging ability of Brevinin-1BW on ABTS radicals showed a weak concentration-dependent effect.

### 2.9. LPS-Binding Assay

**Figure 6:**
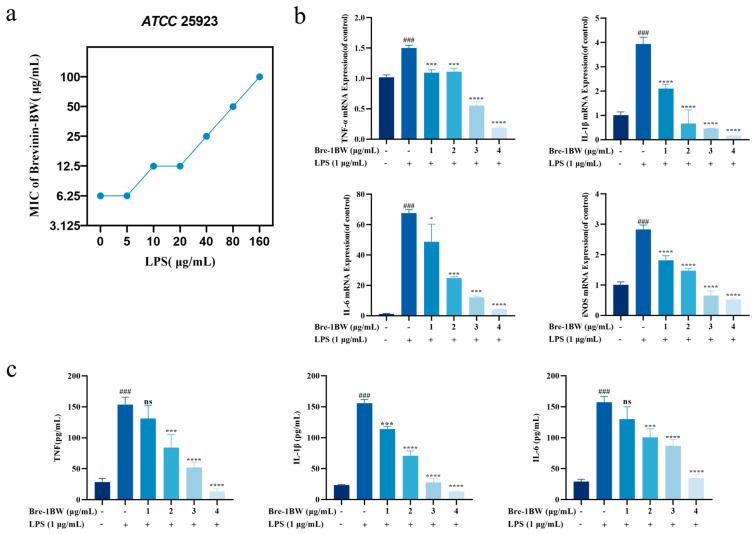
Effect of Brevinin-1BW on the secretion of inflammatory factors in the LPS-induced inflammation model of RAW 264.7 cells. (**a**) Effect of LPS on Brevinin-1BW inhibition of *S. aureus*. (**b**) Effect of Brevinin-1BW on the expression of TNF-α, IL-1β, IL-6, and iNOS mRNA with real-time PCR. (**c**) Analysis of TNF-α, IL-1β, and IL-6 in cell supernatants by ELISA. ### Indicating differences from unprocessed groups, ### (*p* < 0.001). * Represent the differences between the model group and the experimental group **** (*p* < 0.0001), *** (*p* < 0.001), * (*p* < 0.005), ns indicates no significant difference.

We further investigated the interaction of Brevinin-1BW with LPS to reveal the mechanism of action. Figure 6a, the presence of LPS gradually inhibited the antimicrobial efficiency of the peptides in a dose-dependent manner. When the concentration of LPS was higher than 5 μg/mL, the antibacterial activity of Brevinin-1BW against *S. aureus* gradually decreased.

### 2.10. Changes in Bacterial Morphology after Brevinin-1BW Treatment

**Figure 7:**
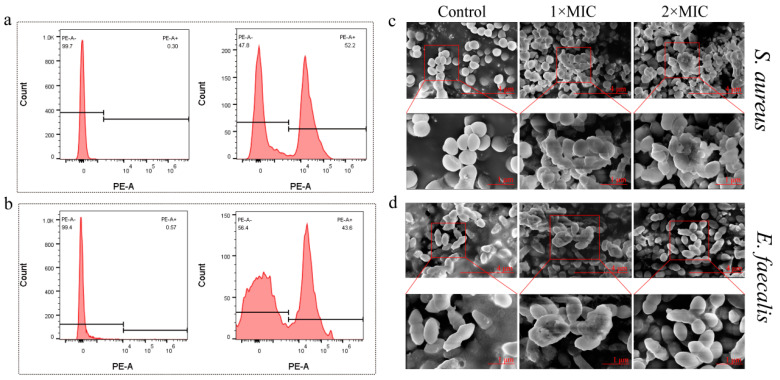
Disruption of *S. aureus* and *E. faecalis* cell membranes by Brevinin-BW analyzed with flow cytometry and SEM. (**a**) *S. aureus* control (left) and treated with 1 × MIC of Brevinin-1BW at room temperature for 2 h (right). (**b**) *E. faecalis* control (left) and treated with 1 × MIC of Brevinin-1BW at room temperature for 2 h (right). (**c**) SEM analysis of *S. aureus* treated with 1 × MIC and 2 × MIC Brevinin-1BW. (**d**) SEM analysis of *E. faecalis* treated with 1 × MIC and 2 × MIC Brevinin-1BW.

Flow cytometry and PI dye were employed to measure the integrity of the bacterial membrane after treatment with the peptide. As shown in Figure 7, *S. aureus* and *E. faecalis* without Brevinin-1BW treatment showed no fluorescence. However, when treated with 1 × MIC, *S. aureus* and *E. faecalis* produced fluorescent signals, with Brevinin-1BW at 1 × MIC causing damage to the bacterial membrane in about 52.2% of *S. aureus* (Figure 7a) and 43.6% of *E. faecalis* (Figure 7b). The results showed that Brevinin-1BW could damage the membranes of both *S. aureus* and *E. faecalis.*

To further confirm that Brevinin-1BW induced cell membrane damage in *S. aureus*, we analyzed the morphological changes of *S. aureus* and *E. faecalis* treated with Brevinin-1BW using SEM. As shown in Figure 7, the surfaces of *S. aureus* and *E. faecalis* in the control group were smooth and intact, while the morphological characteristics of *S. aureus* and *E. faecalis* treated with Brevinin-1BW were wrinkled. In addition, Brevinin-1BW-treated *S. aureus* cells were smaller in size, were more aggregated, and had reduced cell membrane integrity compared to the control group (Figure 7c); *E. faecalis* was more aggregated and had reduced membrane integrity compared to the control group (Figure 7d). There was no significant difference between the two strains treated with 1 × MIC and 2 × MIC.

### 2.11. Anti-Inflammatory Effects of Brevinin-1BW

A lipopolysaccharide (LPS) can induce inflammation in macrophages; therefore, the anti-inflammatory activity of Brevinin-1BW was analyzed using an LPS-induced RAW264.7 cell model. The cytotoxicity of Brevinin-1BW on RAW264.7 cells was previously evaluated to ensure that the concentrations used in subsequent experiments did not affect cell viability. We examined changes in the mRNA levels of inflammatory factors in cell culture supernatants and cells. As shown, the stimulation of RAW 264.7 cells with LPS resulted in the release of large amounts of TNF-α, IL-1β, and IL-6. After treatment with 4 μg/mL Brevinin-1BW, the release of TNF-α, IL-1β, and IL-6 decreased by 87.31, 91.46, and 77.82%, respectively. Real-time PCR results also showed that the mRNA expression levels of TNF-α, IL-6, IL-1β, and iNOS in the LPS stimulation group were decreased by 86.94, 93.72, 95.5, and 81.37%, respectively. In conclusion, Brevinin-1BW significantly inhibited the transcription and expression of inflammatory factors in LPS-stimulated RAW 264.7 cells at non-cytotoxic concentrations.

## 3. Discussion

Antibiotics are the mainstay treatment used for bacterial infections, but due to their pervasive use and misuse, highly antibiotic-resistant pathogenic bacteria continue to emerge. Due to their outstanding antibacterial properties and minimal cytotoxicity, AMPs derived from amphibians are regarded as one of the most promising candidates for developing next-generation antibiotics. AMPs are essential components of the innate immune system and exhibit antibacterial activity against Gram-negative bacteria, Gram-positive bacteria, and fungal pathogens. In contrast to antibiotics, which destroy bacteria by acting on specific targets, AMPs achieve bactericidal activity by rupturing the bacterial membrane and thus have a lower potential to induce resistance. In addition, AMPs serve essential functions in processes pertaining to immunomodulation, anti-inflammation, and cancer prevention.

**Figure 8:**
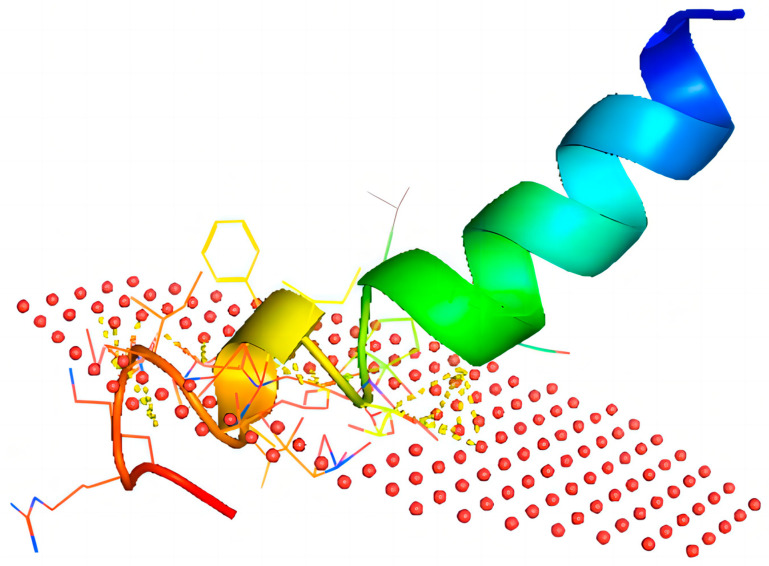
Predicted model for the interaction of Brevinin-1BW with bacterial membranes. Predicted interaction map of Brevinin-1BW with the model membrane (red spheres) generated using the PPM web server, and the results are visualized in PyMOL 2.5 (Schrödinger Inc., CA, USA).

Previously, our team cloned the full length of the cDNA encoding Brevinin-1BW from the skin secretions of *Pelophylax nigromaculatus* but did not fully analyze it. The AMP inhibited the proliferation of several cancer cell lines (A375, A549, and Hela) as well as Gram-positive bacteria (*S. aureus* and *E. faecalis*). Brevinin-1BW was determined to have an α-helical structure with a typical C-terminal loop domain Rana-box, which is similar to the majority of peptides in the Brevinin-1 family [37]. Brevinin-1BW exhibited greater antibacterial activity against clinically resistant *S. aureus* and *E. faecalis* compared to other Brevinin-1 family peptides (Table 5), which demonstrates its potential as an effective agent in the treatment of *S. aureus* infections and wound infections caused by *E. faecalis* worldwide [39,40]. In addition, this antimicrobial peptide also showed good inhibitory effects against multidrug-resistant *S. aureus*, and we will further explore its inhibitory mechanism and further investigate its resistance to clinical multidrug-resistant *S. aureus.* The experimental result shows that it was more effective against Gram-positive bacteria than Gram-negative bacteria, and this is partly attributed to the α-helix structure of AMPs, which modulates interactions with the target membrane. This interaction causes conformational changes to separate hydrophilic residues from hydrophobic residues, and the α-helix is an amphiphilic structure necessary for the target membrane targeting of AMPs [41]. The α-helix forms a bundle in the membrane, with the hydrophobic region interacting with the membrane lipid nucleus and the hydrophilic region pointing inward, thus creating a pore (Figure 8) [42]. On the other hand, its increased bactericidal effect against Gram-positive bacteria compared to Gram-negative bacteria is also partially explained by distinctions in the structure of bacterial cell membranes; Gram-positive bacteria have a denser peptidoglycan layer, whereas Gram-negative bacteria have a thinner and less cross-linked peptidoglycan layer. In addition, Gram-negative bacteria have a relatively denser lipopolysaccharide (LPS) layer compared to their peptidoglycan layer. When attacking Gram-positive bacteria, AMP molecules need only diffuse to the peptidoglycan layer before disrupting the cytoplasmic membrane [43]. However, for Gram-negative bacteria, AMPs must permeate the dense LPS layer before reaching and disrupting the cytoplasmic inner membrane. Herein, flow cytometric assays and SEM experiments demonstrated that Brevinin-1BW rapidly destroys bacteria by penetrating and rupturing the bacterial membrane.

The antibacterial activity of AMPs against bacteria and fungi is determined by the complex interactions between the hydrophilicity or grand average of hydropathy (GRAVY), isoelectric point (pI), cationicity, α-helicity, hydrophobicity, and amphiphilicity [48,49]. Studies on the conformation of AMPs can then be conducted to systematically modify natural molecules or to construct synthetic peptides from inception and determine their structure and biological activity [50]. The goal is to maximize antimicrobial activity and resistance to protein degradation while minimizing toxicity to the host [51]. Researchers have been conducting studies on the structure–function relationship of the Brevinin-1 family of peptides; a family which features relatively high biological activity but whose high hemolytic, high cytotoxic, and low stability characteristics have been limiting their wide biological or clinical applications. The Rana-box structure of the Brevinin-1 peptide family plays several roles: (1) it stabilizes the α-helical structure; (2) the ring structure provides a stable structure for the peptide to resist hydrolysis by proteases, such as carboxypeptidases; (3) it facilitates peptide-induced planar lipid layer membrane conductance and K+ efflux from bacteria; and (4) it provides a net positive charge for some peptides [52]. Some studies have shown that the transfer of the Rana-box structure of the Brevinin-1 family peptides from the C-terminus to the central position significantly reduces the hemolytic activity. Still, other studies have shown that the structure does not affect the antimicrobial activity of certain peptides [53], which suggests that for the position of the Brevinin-1 family Rana-box structure, its deletion is more closely associated with the structural design of the peptide itself. In addition, the hemolytic and antimicrobial activities of peptides are closely related to the length of the α-helix structures within. Second, the hemolytic activity of peptides is related to both the hydrophobicity and the hydrophobic surface [44]. It has also been determined that these two regions are essential for the biological function of the peptide by resynthesizing its analogs through C- and N-terminal amino acid substitution or deletion [38]. Subsequently, we will attempt to enhance the antibacterial activity and stability of Brevinin-1BW by modifying the peptide.

The inflammatory response plays an indispensable role in the process of bacterial infection. Gram-negative bacterial infection produces endotoxin LPS, which activates the macrophage response and induces the release of a variety of cytokines, providing favorable conditions for the occurrence of inflammation [54,55,56]. Through the analysis of the RT-qPCR results, we found that Brevinin-1BW may inhibit the synthesis of pro-inflammatory factors IL-1β, IL-6, and TNF-α, thereby slowing down the process of the inflammatory response to a certain extent. It is possible that Brevinin-1BW attenuates the inflammatory cascade response triggered by LPS by neutralizing LPS and attenuating the stimulation of RAW264.7 cells by LPS. In this sequence, positively charged amino acids, such as arginine (K) and lysine (R), are included. These amino acids can be attracted to the negatively charged part of LPS and promote the charge-to-charge interaction between the sequence and LPS. The sequence also contains some hydrophobic amino acids, such as phenylalanine (F) and alanine (P). These amino acids may interact with the hydrophobic region in LPS and enhance the binding affinity [57]. This not only highlights the potential role of Brevinin-1BW in regulating the immune response, but also provides new ideas for exploring the treatment of inflammation caused by bacterial infections. 

In summary, this study revealed the potential antibacterial and anti-inflammatory mechanisms of a novel antimicrobial peptide. These findings provide important clues for the development of new antimicrobial and anti-inflammatory therapeutic strategies, especially in the face of increasing antibiotic resistance. However, further experimental studies and clinical trials will be key to validate and gain a deeper understanding of these mechanisms.

## 4. Materials and Methods

### 4.1. Identification and Synthesis of Brevinin-1BW

The amino acid sequence of the peptide Brevinin-1BW (FLPLLAGLAASFLPTIFCKISRKC) was determined and provided by previous investigators in the laboratory, and the peptide was provided by Gill Biochemical (Shanghai, China) Co., Ltd. A ChromCore 120 C18 5 μm column, equilibrated with water/acetonitrile solution containing 0.1% trifluoroacetic acid (46.0/54.0) at a flow rate of 1 mL/min, was purified to near homogeneity with RP-HPLC. A linear gradient was used to increase the acetonitrile concentration to 71% in 25 min. The purity of the final synthesized peptide was >95%, identified by electrospray mass spectrometry.

### 4.2. Structural Analysis and Sequence Alignment

The obtained peptide sequences were analyzed using bioinformatic techniques. The chemical structure of Brevinin-1BW was analyzed by an online web server (https://www.allpeptide.com/canshu.html, accessed on 29 June 2023). The helical wheel structure and secondary structure were analyzed using the HeliQuest analysis tool (http://heliquest.ipmc.cnrs.fr/, accessed on 28 March 2023) and the I-TASER online server (https://zhanggroup.org/I-TASSER/registration.html, accessed on 16 June 2023) for predictive analysis. The 3D structure was predicted using the AlphaFold2 server (https://colab.research.google.com/github/sokrypton/ColabFold/blob/main/AlphaFold2.ipynb, accessed on 28 June 2023).

### 4.3. Biological Materials and Reagents

The bacterial strains *Enterococcus faecalis* (ATCC 29212), *Staphylococcus aureus* (ATCC 25923), *Staphylococcus saprophyticus* (ATCC BAA750), multidrug-resistant *Staphylococcus aureus* (MDR ATCC 29213), *Enterobacter hormaechei* (ATCC 700323), *Pseudomonas aeruginosa* (ATCC 27853), *Klebsiella pneumoniae* (ATCC 700603), and multidrug-resistant *Escherichia coli* (MDR ATCC 35218) were obtained from the First People’s Hospital in An Ning, Yunnan Province. The bacterial strains *Staphylococcus epidermidis* (CI 1607BL1D39), *Staphylococcus haemolyticus* (CI 1607BLD590), and *Citrobacter koseri* (CI 1611SED223) were provided by the Yunnan Molecular Diagnostic Center. Human lung cancer cells A549, human osteosarcoma cells U-2OS, human hepatoma cells HePG2, human immortalized epidermal cells HaCaT, human umbilical vein endothelial cells HUVEC, human embryonic kidney cells 293T, human malignant melanoma cells A375, human colon cancer cells HCT116, human cervical cancer cells Hela, human foreskin fibroblast cells HFF, and mouse mononuclear macrophages RAW264.7 were all obtained from the Research Center of Molecular Medicine of Yunnan Province.

### 4.4. Antibacterial Activity Analysis

The minimum inhibitory concentration (MIC) of the Brevinin-1BW was measured in 96-well microtiter plates according to the Clinical and Laboratory Standards Institute (CLSI). The activated strains were treated with Mueller Hinton Agar (MHA) solid medium. Single colonies were picked and expanded to the logarithmic growth phase, and the solution was diluted to 2 × 10^6^ CFU/mL using the McFarland turbidity standard. The MIC was determined with the two-fold dilution method. Briefly, 50 μL of Mueller Hinton (MH) medium was added to a 96-well plate in advance. Then, 50 μL of AMP solution was added to the first well, and 50 μL was pipetted into the next well after mixing using a row gun, while 50 μL was discarded from the last well. Final concentrations of Brevinin-1BW were 100, 50, 25, 12.5, 6.25, and 3.125 μg/mL. A 50 μL volume of the diluted bacterial solution was added, and the final concentration of the bacterial solution was 1 × 10^6^ CFU/mL. This was then placed into a 37 °C incubator for 18 h until the next day to observe the degree of turbidity of the 96-well plate and measure the absorbance of the wells at 600 nm with a multifunctional microplate reader (SpectraMax iD5, Molecular Devices, Sunnyvale, CA, USA). The absence of turbidity and the measured concentration of absorbance being less than the opposing group was taken as the MIC. The positive control used ampicillin at the same final concentration as Brevinin-1BW, the negative control used 50 μL of MH medium mixed with 50 μL of 2 × 10^6^ CFU/mL bacterial solution, and the blank contained only 100 μL of MH medium. Three replicate wells were set up for each experiment, and each experiment was repeated three times. The MIC and all concentrations above the MIC of bacterial solution coated on MHA solid medium were placed into 37 °C incubator incubation for 18 h. The lowest concentration resulting in no colony growth was taken as the minimum bactericidal concentration (MBC).

### 4.5. Time-Killing Assay of Brevinin-1BW against S. aureus and E. faecalis

The concentration of *S. aureus* (ATCC 25923) and *E. faecalis* (ATCC 29212) in the logarithmic growth phase was adjusted to 1 × 10^6^ CFU/mL. A 100 μL volume of diluted bacterial solution and 100 μL of Brevinin-1BW solution were incubated together. The final concentration of Brevinin-1BW was 1 × MIC, while an equal volume of sterile water was added to the blank control. The incubation was carried out at 37 °C in a 110 rpm shaker. Then, 100 μL of the mixture was added with 2 μM dead cell green cytosine SYTOX Green (Alphabio, Tianjin, China) at 0 min, 30 min, 60 min, 90 min, and 120 min, respectively, and then incubated for 8 min at room temperature protected from light. The resulting fluorescence absorbance was measured on a multifunctional microplate reader at an excitation wavelength of 525 nm and emission wavelength of 485 nm. The fluorescence absorbance was recorded as *A*_0_ for the blank group and *A*_1_ for the experimental group. The experiment was repeated three times.

$$Bacterial\;Survival\;Rate\left(\%\right)=[1-\frac{\left(A_{1}-A_{0}\right)}{A_{1}}]\times 100$$

### 4.6. Antibiofilm Assay

Biofilm inhibition of Brevinin-1BW was determined using a 96-well plate assay in which 100 µL of Brevinin-1BW peptide solution (0.5–8 × MIC) was mixed with 100 µL of *S. aureus* (ATCC 25923) suspension (5 × 10^5^ CFU/mL) in MH medium and incubated for 24 h at 37 °C. The plates were washed three times with sterile PBS and fixed with methanol for 0.5 h. After drying, the cells were stained with 1% crystal violet solution for 0.5 h. They were washed three times with deionized water and dissolved in 100 µL absolute ethanol. Absorbance was measured at 600 nm. Experiments were repeated four times independently.

Biofilm clearance of Brevinin-1BW was determined using a 96-well plate assay by first inoculating 100 µL of *S. aureus* bacterial suspension (5 × 10^5^ CFU/mL) and incubating for 24 h at 37 °C. They were washed three times with sterile PBS. Peptide solution (0.5 to 8 × MIC) or 100 µL of MH medium was added. After 24 h of incubation, the cells were washed three times with sterile PBS. Methanol was added and fixed for 0.5 h and dried aseptically. A 1% crystal violet solution was added for 0.5 h and washed three times with sterilized deionized water. Absolute ethanol dissolved the biofilm. Absorbance was measured at 600 nm. Experiments were repeated four times independently.

### 4.7. Stability Analysis of Brevinin-1BW

The temperature, serum, and pH stability of antimicrobial peptide Brevinin-1BW was investigated. *S. aureus* (ATCC 25923) was used to determine its stability. The antimicrobial peptide solution was prepared and placed at −20 °C, 4 °C, 37 °C, 60 °C, and 100 °C for 1 h. The MIC was measured with 96-well plate method to detect the effect of temperature. After incubating the Brevinin-1BW solution with 0%, 5%, and 10% fetal bovine serum (TransGen Biotech, Beijing, China) at 37 °C for 1 h, the MIC was determined with a 96-well plate assay to test the serum stability of AMP. The pH stability was achieved by inoculating *S. aureus* into MH medium and culturing them with shaking to reach the logarithmic growth phase. This was then diluted to 10^6^ CFU/mL. Brevinin-1BW was incubated in PBS at various pH values, pH = 2 or 12, for 1 h at 37 °C. The minimal inhibitory concentrations (MIC) of the peptides were then determined. All the above experiments were repeated more than three times.

### 4.8. Hemolysis Assays

A hemolytic assay of Brevinin-1BW was conducted using sheep erythrocytes (Solarbio, Beijing, China) at a concentration of 2% (*v*/*v*) of erythrocyte suspension. First, 20 µL of peptide solution was mixed with 180 μL of 2% sheep erythrocyte suspension to reach a final peptide concentration of 3.125–100 μg/mL, which was then incubated at 37 °C for 2 h. PBS was used as a negative control, and 0.1% Triton X-100 was used as a positive control. After centrifugation at 1000 rpm for 15 min, the absorbance of the supernatant was analyzed using a multifunctional enzyme standardizer with the wavelength set at 570 nm. The experiment was repeated three times. The peptide hemolysis rate was calculated according to the following equation, where *A*_0_ denotes the absorbance of the negative control group, *A*_1_ denotes the absorbance of the positive control group, and *A_2_* represents the absorbance of the experimental group.

$$Haemolysis\left(\%\right)=\frac{\left(A_{0}-A_{2}\right)}{\left(A_{0}-A_{1}\right)}\times 100$$

### 4.9. Cytotoxicity Assays

Medium was prepared to contain 10% FBS (TransGen Biotech, China) and 1% penicillin-streptomycin (Biosharp, Guangzhou, China). A549, U-2OS, HePG2, HaCaT, HUVEC, and 293T cells were cultured in DMEM medium (Gibco, New York, NY, USA). A375, HCT116, Hela, and RAW264.7 cells were cultured in RPMI 1640 medium (Gibco, New York, NY, USA). The cytotoxicity of Brevinin-1BW was determined using the Cell Counting Kit-8 as follows: detached the cells from cell culture flasks with 0.25% trypsin, counted with a hemocytometer plate and then prepared into a cell suspension containing 5 × 10^3^ cells/mL. Then, 90 μL of the cell suspension was inoculated into a 96-well plate and incubated at 37 °C in a 5% CO_2_-humidified atmosphere for 24 h. A 10 μL volume of the peptide was subsequently added to each well and co-incubated with the cells for 24 h. Then, 10 μL of CCK8 (Abbkine, Wuhan, China) was added to each well and incubated for a further 2 h. The absorbance of each well at 450 nm was measured using a multifunctional enzyme marker. The final concentrations of peptides were 100, 50, 25, 12.5, 6.25, and 3.125 μg/mL, respectively. The experiments were also performed with wells containing only medium and cells as the negative control group, which was recorded as *A*_0_, while the group containing medium, cells, and peptides served as the experimental group, which was recorded as *A*_1_. Three replicate wells were set up for each experiment, and each experiment was repeated three times. The formula for calculating the cell viability is shown below.

$$Cell\;Viability\left(\%\right)=\frac{A_{0}-A_{1}}{A_{0}}\times 100$$

### 4.10. Antioxidant Activity Analysis

ABTS^+^ scavenging capacity: A 7 mM ABTS (Med Chem Express, NJ, USA) solution and 140 mM potassium persulfate (Aladdin, Shanghai, China) solution were prepared. A 5 mL volume of ABTS was taken and mixed with 88 μL of potassium persulfate before being placed in the dark at room temperature for 14 h to form the ABTS radical stock solution. The ABTS radical stock solution was then diluted with 95% ethanol by volume to reach an OD_734 nm_ = 0.7. Then, 10 μL of Brevinin-1BW solution was mixed with 50 μL of ABTS radical stock solution, and the resulting absorbance was measured at 734 nm for 6 min at room temperature, which was recorded as *A*_1_. The absorbance of 95% ethanol was measured as *A*_2_. The same concentration of vitamin C was used as the positive control. The clearance was calculated according to the following equation:

$${ABTS}^{+}\;Clearance\;Rate\left(\%\right)=\frac{\left(A_{2}-A_{1}\right)}{A_{2}}\times 100$$

DPPH^+^ scavenging capacity: A 30 mL volume of DPPH^+^ (Rhawn, Shanghai, China) solution was prepared with anhydrous ethanol and adjusted to reach an OD_517 nm_ = 0.7. Then, DPPH^+^ alcohol solution and water were added in a volume ratio of 1:1 as the blank control, while Brevinin-1BW solution and anhydrous ethanol served as the negative control; their absorbances were recorded at 517 nm as *A*_0_ and *A*_1_, respectively. The Brevinin-1BW and DPPH solutions were mixed in equal volumes as the experimental group, incubated at 37 °C in the dark for 30 min, and then measured for the absorbance as *A*_2_. Vitamin C was used as the positive control. Its clearance rate was calculated according to the following formula:

$${DPPH}^{+}\;Clearance\;Rate\left(\%\right)=\left[1-\frac{\left(A_{2}-A_{1}\right)}{A_{0}}\right]\times 100$$

### 4.11. LPS-Binding Assay

The lipopolysaccharide-binding assay was performed using the checkerboard method. Dissolve and prepare LPS (Solarbio, Beijing, China) and Brevinin-1BW with Muller Hinton (MH), add LPS with a final concentration of 0–160 μg/mL along the X-axis, and add 100–0 μg/mL Brevinin-1BW along the Y-axis. Then, an additional 10^5^ CFU of *S. aureus* was added to bring the final volume to 200 μL. It was incubated at 37 °C for 18 h, measure the OD 600 value, and the MIC value at different LPS concentrations was recorded. The experiment was repeated three times.

### 4.12. Membrane Permeability and Morphology Change Observation

A 3 mL volume of *E. faecalis* (ATCC 29212) and *S. aureus* (ATCC 25923) bacterial solution, respectively, was taken at the logarithmic growth stage and centrifuged at 5000 rpm for 10 min (NACHT, Heidelberg, Germany), and then, the resulting colony precipitate was washed with PBS three times for 10 min each time. The resulting concentration of each bacterial solution was adjusted to 1 × 10^6^ CFU/mL. Then, 1 mL of adapted bacterial solution was incubated with Brevinin-1BW at 37 °C for 2 h so that the final concentration of Brevinin-1BW was 1 × MIC and 2 × MIC, respectively. Sterile water only was added to the negative control group. At the end of the incubation period, PI staining solution (Biosharp, Guangzhou, China) was added to each tube to reach a final concentration of 30 μM and was then incubated for 15 min at room temperature protected from light. No blank group was included. Detection was performed using a flow cytometer (BD Biosciences, San Jose, CA, USA).

*E. faecalis* and *S. aureus* were cultured to reach the logarithmic growth phase, after which each bacterial solution concentration was adjusted to 1 × 10^6^ CFU/mL. Then, 1 mL of each adjusted bacterial solution was incubated with Brevinin-1BW at final concentrations of 1 × MIC and 2 × MIC at 37 °C for 2 h. For the negative control, only an equal volume of sterile water was added and centrifuged at 5000 rpm for 10 min. The resulting precipitate was washed three times with PBS for 10 min each time. Then, 500 μL of electron microscope fixative (Servicebio, Wuhan, China) was added to each tube and fixed overnight at 4 °C. Following this, each solution was centrifuged at 5000 rpm for 10 min before being washed three times with PBS for 10 min each time. The precipitate was sequentially incubated with 100% ethanol and 100% ethanol: acetone (1:1) before 100% acetone was used to dehydrate the samples. Finally, the samples were dried overnight in a freeze-dryer. The samples were then sprayed, plated, and imaged with SEM.

### 4.13. Proinflammatory Cytokine Level

RAW 264.7 mouse macrophages were cultured in DMEM medium (Gibco, New York, NY, USA). containing 10% FBS (TransGen Biotech, Beijing, China) and 1% penicillin-streptomycin (Biosharp, Guangzhou, China) and were seeded into 6-well plates at a cell density of 10^5^ cells/mL. After cell adhesion, 1 μg/mL LPS (Solarbio, Beijing, China) and different concentrations of Brevinin-1BW (0, 1, 2, 3, and 4 μg/mL) were incubated for 24 h. Culture supernatants were collected, and the contents of TNFα, IL-6, and IL-1β were determined with enzyme-linked immunosorbent assay (BOSTER, Wuhan, China). All cells were washed twice with precooled PBS, total cellular RNA was extracted using the RNAsimple Total RNA Extraction kit (TIANGEN, Beijing, China), and total RNA concentration and purity were determined using a Thermo Fisher NanoDropOne nucleic acid protein spectrophotometer. HiScript II 1st Strand cDNA Synthesis Kit (Vazyme, Nanjing, China) synthesized cDNA, and we performed real-time PCR using Hieff^®^ qPCR SYBR Green Master Mix (No Rox) (YEASEN, Shanghai, China). The primer sequences for Beta-actin (β-Actin), interleukins (IL-6, IL-1β), tumor necrosis factor (TNF-α), and nitric oxide synthase (iNOS) are shown in Table 6. Cells incubated without peptide and/or LPS were used as negative control. All experiments were repeated three times.

### 4.14. Data Analysis

GraphPad Prism9.4.1 software (San Diego, CA, USA) was used to analyze all biological activity evaluation experimental data. The measurements were analyzed using two-way ANOVA and Dunnett’s multiple comparison test. Error bars in the graphs indicate the standard error of the mean (SEM) for experiments performed in more than three sets of replicates. Significant differences are shown by **** (*p* < 0.0001), *** (*p* < 0.001), ** (*p* < 0.001), and * (*p* < 0.005).

## Figures and Tables

**Table 1:** Structural parameters of Brevinin-1BW.

_1_FLPLLAGLAASFLPTIFCKISRKC_24_		
Physico-chemical properties	Polar residues + GLY	Nonpolar residues
Hydrophobicity <H>	Polar residues + GLY (n/%)	Nonpolar residues (n/%)
0.838	7/29.17	17/70.83
Hydrophobic moment <µH>	Uncharged residues + GLY	Aromatic residues
0.256	SER 2, THR 1, GLY 1	PHE 3
Net charge z	Charged residues	Special residues
3	LYS 2, ARG 1	CYS 2, PRO 2
Hydrophobic face: I A L I L A C F A		

**Table 2:** Antimicrobial activity of Brevinin-1BW.

Bacterial Strains	Brevinin-1BW		Ampicillin
	MIC (μg/mL)	MBC (μg/mL)	MIC (μg/mL)
Gram-positive bacteria			
*S. aureus* (ATCC25923)	6.25	6.25	3.125
*E. faecalis* (ATCC29212)	3.125	12.5	3.125
*S. saprophytics* (ATCCBAA750)	6.25	12.5	3.125
*S. aureus* (MDR ATCC29213)	6.25	25	>100
*S. epidermidis* (CI 1607BL1D39)	12.5	25	3.125
*S. haemolyticus* (CI 1607BLD590)	12.5	25	>100
Gram-negative bacteria			
*E. coli* (ATCC25922)	100	>100	100
*E. hormaechei* (ATCC700323)	100	>100	>100
*P. aeruginosa* (ATCC27853)	100	>100	>100
*K. pneumoniae* (ATCC700603)	>100	>100	>100
*E. coli* (MDR ATCC35218)	>100	>100	>100
*C. koseri* (CI 1611SED223)	50	>100	>100

CI, clinically isolated strain; MDR, multidrug-resistant standard strains. The results were obtained from 9 replicates of 3 independent experiments. MIC is the lowest concentration at which the bacteria cannot grow. MBC is the lowest concentration without any bacterial colonization. All strains used ampicillin as a positive control.

**Table 3:** Effect of temperature, serum, and pH on Brevinin-1BW.

	Conditions	MIC (µg/mL) *		Conditions	MIC (µg/mL) *
Temperature	−20 °C	6.25	Fetal bovine serum	0	6.25
	4 °C	6.25		5%	6.25
	37 °C	6.25		10%	12.5
	60 °C	6.25	pH	2	6.25
	100 °C	6.25		12	6.25

* *S. aureus* was used to detect the antimicrobial activity of the peptide. The results represent the mean values of three independent experiments performed in triplicate.

**Table 4:** The CC_50_ * values of Brevinin-1BW on different cells.

Cancer Cells	CC_50_ (µg/mL)	Non-Cancer Cells	CC_50_ (µg/mL)
A375	23.79 ± 0.80	293T	28.34 ± 1.08
A549	27.64 ± 0.41	HUVEC	43.62 ± 0.77
Hela	37.62 ± 0.91	HaCaT	36.84 ± 1.44
HePG2	25.42 ± 0.49	HFF	33.99 ± 0.48
U-2OS	39.20 ± 0.40	RAW264.7	32.33 ± 0.47
HCT116	35.44 ± 0.47		

* Cytotoxic concentration to kill 50% of cells (CC_50_) was determined from dose-response curves ([inhibitor] vs. response with variable slope (four parameters), n ≥ 3, ±SE).

**Table 5:** Comparison of MICs of Brevinin-1BW and related AMPs congeners against specific microorganisms.

Peptide Name	GRAVY	pI	MIC (µM)		
			*S. aureus*	MRSA/MDR *S. aureus*	*E. faecalis*
Brevinin-1BW (FLPLLAGLAASFLPTIFCKISRKC)	1.192	9.50	2.38	2.38	1.19
Brevinin1TSa (FLGSIVGALASALPSLISKIRN)	1.073	11.0	25	25	50
Brevinin-1ISa (FLPGVLRLVTKVGPAVVCAITRNC)	1.104	9.70	3.1	3.1	—
Brevinin-1JDa (FLPAVIRVAANVLPTVFCAISKKC)	1.279	9.50	6	13	—
Brevinin-1JDc (FLPAVLRVAAKVVPTVFCLISKKC)	1.333	9.85	6	3	—
Brevinin-1GHa (FLGAVLKVAGKLVPAAICKISKKC)	1.054	9.90	2	4	8
Brevinin-1GHd (FLGALFKVASKLVPAAICSISKKC)	1.108	9.70	2	4	—

The sequences and MICs of other antimicrobial peptides come from publications [44,45,46,47]. The “—” indicates that the strain was not tested in the references.

**Table 6:** Primer sequences (mouse) of inflammatory factors.

Gene	Forward Primer (5′ → 3′)	Reverse Primer (5′ → 3′)
β-Actin	TATCGGACGCCTGGTTA	TGTGCCGTTGAACTTGC
TNF-α	GTCAACCTCCTCTCTGCCAT	CCAAAGTAGACCTGCCCAGA
IL-1β	CCAGGATGAGGACATGAGCA	CGGAGCCTGTAGTGCAGTTG
IL-6	TCCATCCAGTTGCCTTCTTG	AAGCCTCCGACTTGTGAAGTG
iNOS	CATGCTACTGGAGGTGGGTG	CATTGATCTCCGTGACAGCC

## Data Availability

Data are contained within the article.
